# Serum and Adipose Tissue mRNA Levels of ATF3 and FNDC5/Irisin in Colorectal Cancer Patients With or Without Obesity

**DOI:** 10.3389/fphys.2018.01125

**Published:** 2018-09-10

**Authors:** Huijuan Zhu, Meijuan Liu, Nianrong Zhang, Hui Pan, Guole Lin, Naishi Li, Linjie Wang, Hongbo Yang, Kemin Yan, Fengying Gong

**Affiliations:** ^1^Key Laboratory of Endocrinology of National Health Commission, Department of Endocrinology, Peking Union Medical College Hospital, Chinese Academy of Medical Sciences, Peking Union Medical College, Beijing, China; ^2^Department of General Surgery, Peking Union Medical College Hospital, Chinese Academy of Medical Sciences, Peking Union Medical College, Beijing, China

**Keywords:** activating transcription factor 3, fibronectin type III domain-containing protein 5/irisin, colorectal cancer, obesity, adipose tissue

## Abstract

**Objectives:** To explore the activating transcription factor 3 (ATF3) and fibronectin type III domain-containing protein 5 (FNDC5)/irisin protein levels in serum and mRNA levels in subcutaneous and visceral white adipose tissue (sWAT and vWAT) in normal-weight (NW) and overweight/obese (OW/OB) patients with colorectal cancer (CRC).

**Methods:** 76 CRC patients and 40 healthy controls were recruited. Serum ATF3 and irisin levels were detected by using ELISA kits, and the mRNA expression levels in sWAT and vWAT were measured by performing RT-qPCR.

**Results:** The serum ATF3 levels were greater by 37.2%, whereas the irisin levels were lower by 23.3% in NW+CRC patients compared with those in healthy controls. CRC was independently associated with both ATF3 and irisin levels. The probability of CRC greater by 22.3-fold in individuals with high ATF3 levels compared with those with low ATF3 levels, whereas the risk of CRC in subjects with high irisin levels was lower by 78.0% compared to the risk in those with low irisin levels after adjustment for age, gender, BMI, and other biochemical parameters. Serum ATF3 and irisin could differentiate CRC patients from controls with receiver operating characteristic (ROC) curve areas of 0.745 (95% CI, 0.655–0.823) and 0.656 (95% CI, 0.561–0.743), respectively. The combination of ATF3 and irisin exhibited improved diagnosis value accuracy with ROC curve areas of 0.796 (95% CI, 0.710–0.866) as well as 72.6% sensitivity and 80.0% specificity.

**Conclusion:** Increased ATF3 and reduced irisin levels were observed in sera from CRC patients. Individuals with high ATF3 and low irisin levels were more likely to have CRC. ATF3 and irisin represent potential diagnostic biomarkers for CRC patients.

## Introduction

According to the annually updated nationwide statistics from the National Central Cancer Registry of China (NCCRC), the incidence of colorectal cancer (CRC) ranked as the fourth predominant cancer in men and the third in women. It is estimated that 347,900 individuals have been diagnosed with CRC, and 164,900 people died of CRC in 2013 in China (Chen et al., 2017). Furthermore, both the incidence and mortality trends of CRC had significantly increased at the rates of 2.5 and 1.3%, respectively, from 2000 to 2013 in China (Chen et al., 2017). Clearly, CRC has become a serious public health problem; hence, CRC-related mechanisms are currently a research area of increased interest.

Although the mechanisms of CRC are multifactorial and complex, it is well accepted that obesity plays a critical role in the onset and progression of CRC. A recently updated review by Bardou et al. (2013) reported that obesity was associated with a 30–70% increased risk of CRC compared with that in normal-weight people. Adipose tissue, which was previously perceived to be a storage reservoir of fat, is now recognized as an active endocrine organ that secretes various bioactive adipokines (Prieto-Hontoria et al., 2011). The dysregulated secretion of adipokines in obese conditions contributes considerably to the pathogenesis of CRC (van Kruijsdijk et al., 2009; Prieto-Hontoria et al., 2011; Perez-Hernandez et al., 2014; Zhu et al., 2018).

Activating transcription factor 3 (ATF3) and irisin are two types of novel adipocytokines. ATF3, one of the smallest members of the ATF/CREB family of transcription factors, is a recently identified adipokine that plays an important role in maintaining genetic integrity and cellular homeostasis under stress conditions (Tsai et al., 2013). Previous studies on human breast cancer (Wolford et al., 2013), lung cancer (Li et al., 2017), skin cancer (Hao et al., 2013), and CRC (Wu et al., 2014; Yan et al., 2017) have demonstrated increased ATF3 expression in cancer tissues/cells compared with that in normal tissues/cells. Furthermore, studies by Li et al. (2017) also demonstrated that high ATF3 expression in tumor tissues was positively correlated with advanced tumor grade, lymph node metastasis, and reduced overall survival. These data underscore the important role of ATF3 in the pathogenesis and progression of human cancers.

Irisin is produced by the proteolytic cleavage of the fibronectin type III domain-containing protein 5 (FNDC5) (Bostrom et al., 2012) and plays a pivotal role in the occurrence and development of obesity, diabetes, non-alcoholic fatty liver disease, and other metabolic diseases (Arias-Loste et al., 2014; Shelbaya et al., 2017; Suk et al., 2018). However, recent studies conducted by Kuloglu et al. (2016) demonstrated that irisin immunoreactivity was significantly increased in human breast cancer tissues compared with that in healthy breast tissues, indicating that irisin may also play a critical role during carcinogenesis. Moreover, Provatopoulou et al. (2015) found that the serum levels of irisin in breast cancer patients were significantly reduced compared with those in healthy controls, and one unit increase in irisin levels resulted in an approximately 90% reduction in the probability of breast cancer. Further ROC curve analysis demonstrated that irisin could effectively discriminate breast cancer patients with 62.7% sensitivity and 91.1% specificity at a cut-off point of 3.21 μg/mL, suggesting that serum irisin may serve as a potential biomarker for the early detection of breast cancer (Provatopoulou et al., 2015).

Although the above studies have demonstrated that changes in ATF3 and irisin levels occur in human cancer tissues, such as breast cancer (Wolford et al., 2013), lung cancer (Li et al., 2017), skin cancer (Hao et al., 2013), and CRC (Wu et al., 2014; Yan et al., 2017) tissues, little is known about circulating and adipose tissue expression levels of ATF3 and irisin in CRC patients. Therefore, the present study was undertaken to explore the roles of ATF3 and irisin in human CRC by determining serum and mRNA levels in subcutaneous and visceral white adipose tissues (sWAT and vWAT) of ATF3 and irisin among normal-weight (NW) and overweight/obese (OW/OB) CRC patients. The relationship between these two factors and CRC and their potential diagnostic value for CRC screening were also investigated.

## Materials and Methods

### Subjects

In total, 76 patients with CRC (38 colon cancer cases and 38 rectal cancer cases) were recruited from the department of General Surgery in Peking Union Medical College Hospital. All patients were pathologically confirmed to have colon/rectal cancer. The body mass index (BMI) score of each patient was calculated by dividing the individual’s body weight in kilograms by the square of the individual’s height in meters. People with a BMI < 18 kg/m^2^ and those with acute inflammatory diseases, chronic rheumatic diseases, or other malignant neoplasms were not included in the present study. In addition, 40 healthy subjects (18 kg/m^2^ < BMI < 25 kg/m^2^) who served as controls were collected from the health examination center in the outpatient department during the same period. CRC patients were further divided into the NW+CRC group (18 kg/m^2^ < BMI < 25 kg/m^2^, *n* = 42) and OW/OB+CRC group (BMI ≥ 25 kg/m^2^, *n* = 34). Participants in our study provided informed consent, and the experimental processes were performed with the approval of the ethics committee of Peking Union Medical College Hospital (No. S-364).

### Blood and Tissue Sample Collection and Processing

Peripheral venous blood samples from all patients and healthy controls were collected preoperatively after overnight fasting. Then, the serum samples were immediately centrifuged at 3000 *g* for 10 min, aliquoted, and frozen at -80°C. In addition, sWAT and vWAT were obtained from nine NW+CRC and nine OB+CRC patients during the surgical process. The adipose tissue samples were immediately collected in sterile tubes, frozen in liquid nitrogen, and preserved at -80°C until they were assayed.

### Serum Biochemical Assays and Adipokine Measurements

Serum fasting blood glucose (FBG), triglyceride (TG), total cholesterol (TC), high-density lipoprotein cholesterol (HDL-C), and low-density lipoprotein cholesterol (LDL-C) levels were measured in our clinical laboratory by using routine automated laboratory methods. ATF3 and irisin protein levels were determined using commercially available enzyme-linked immunosorbent assay (ELISA) kits (USCN Life Science Inc., Wuhan, China) by following the manufacturer’s instructions. The coefficients of intra- and interassay variation were 1.8 and 4.5%, respectively, for ATF3 and 1.2 and 13.9%, respectively, for irisin.

### Total RNA Preparation and Reverse Transcription Quantitative PCR (RT-qPCR)

Total RNA from sWAT and vWAT was extracted by using the E.Z.N.A Total RNA Kit I (Omega Bio-tek, United States). The RNA quantity was evaluated using a NanoDrop 2000C (Thermo Forma, United States). The OD260/280 ratios of all samples were between 1.9 and 2.1. Then, the reverse transcription reaction was performed on an ABI 7500 PCR instrument (Applied Biosystems, CA, United States) at 37°C for 60 min by using 1.0 μg RNA, 1.0 μL OmniScript Reverse Transcriptase (Qiagen Lot 205111, Hilden, Germany), and 10 U RNase inhibitor together with an oligo(dT) primer provided by Promega (Madison, WI, United States). Each gene was measured in duplicate in a final volume of 20 μL. The amplification was performed following a standard thermal cycler protocol, and each gene exhibited specific amplification verified by the dissociation curve. The sequences of the primers were as follows: β-*actin*, forward 5′-TCCCTGGAGAAGAGCTAC G-3′, reverse 5′-GTAGTTTCGTGGATGCCACA-3′; *ATF3*, forward 5′-CCTCTGCGCTGGAATCAGTC-3′, reverse 5′-TTCTTTCTCGTCGCCTCTTTTT-3′; *FNDC5*, forward 5′-GCCTGTGCTCTTCAAGACCC-3′, reverse 5′-CATGAACAGGACCAC GACGA-3′. The mean Ct value for each gene was used for data analysis. The results are represented as fold changes of the Ct score relative to the housekeeping gene β-*actin* by using the 2^-ΔΔCt^ formula (Livak and Schmittgen, 2001).

### Statistical Analyses

Data are presented as the mean ± standard deviation (SD) when normally distributed or as the median (25th–75th percentile) when data are skewed. The normality of the distribution was evaluated using the Shapiro–Wilk test. Based on the data distribution, either the independent sample *t*-test or the Mann–Whitney *U* test was used for the comparison of variables between two groups. Bivariate correlation coefficients were applied to evaluate the correlations between serum ATF3 or irisin levels and other anthropometric characteristics and biochemical variables. The odds ratio (OR) and 95% confidence intervals (CI) of serum ATF3 and irisin for CRC were estimated using univariate and multivariate logistic regression analyses. Cut-off point analysis, namely, the largest distance from the diagonal line of the receiver operating characteristic curve (ROC) (sensitivity × [1 – specificity]), was used to detect the optimal value of serum ATF3 and irisin levels that could differentiate CRC patients from healthy controls. All statistical analyses were performed using SPSS 20.0 for Windows (SPSS Inc., Chicago, IL, United States). *P* < 0.05 was considered significant.

## Results

### Baseline Characteristics of All Participants in NW+CRC, OW/OB+CRC, and Control Groups

Baseline characteristics of all participants are summarized in **Table 1**. As we expected, the body weight, BMI, systolic blood pressure (SBP), and TG in the OW/OB+CRC group were significantly increased compared with those in the NW+CRC group (all *P* < 0.05). In addition, significantly increased age but lower HDL-C levels were observed in NW+CRC patients compared with those in controls (both *P* < 0.05) as shown in **Table 1**.

**Table 1:** General clinical and laboratory parameters of participants in controls, NW+CRC and OW/OB+CRC groups.

Variables	Controls (*n* = 40)	Cancer (*n* = 76)		
		
		NW+CRC (*n* = 42)	OW/OB+CRC (*n* = 34)	All CRC (*n* = 76)
Gender (M/F)	30/10	29/13	19/15	48/28
Age (y)	61.0 (59.0–66.0)	67.9 ± 9.4*^a^*	69.0 (59.0–78.3)	68.0 (62.0–76.0)*^a^*
Height (cm)	166.5 ± 8.0	166.5 ± 6.3	165.9 ± 9.3	166.3 ± 7.8
Body weight (kg)	63.2 ± 7.8	61.2 ± 6.3	79.3 ± 11.7*^b^*	66.8 (60.0–76.4)*^a^*
BMI (kg/m^2^)	22.5 ± 1.9	22.0 ± 1.6	28.2 (26.5–30.3)*^b^*	24.2 (22.0–27.7)*^a^*
SBP (mmHg)	121.3 ± 9.8	123.4 ± 15.4	131.5 ± 12.4*^b^*	127.0 ± 14.6*^a^*
DBP (mmHg)	74.7 ± 6.9	73.5 ± 9.8	75.7 ± 9.7	74.5 ± 9.8
FBG (mmol/L)	5.04 ± 0.43	5.20 (4.80–5.55)	5.50 (4.95–6.15)	5.30 (4.80–5.80)*^a^*
TC (mmol/L)	4.87 (4.32–5.26)	4.62 ± 0.75	5.04 ± 1.16	4.81 ± 0.97
TG (mmol/L)	1.23 ± 0.50	1.00 (0.87–1.60)	1.48 (1.25–1.79)*^b^*	0.94 (1.30–1.66)
HDL-C (mmol/L)	1.12 (1.32–1.64)	1.13 ± 0.26*^a^*	1.04 ± 0.18	1.09 ± 0.23*^a^*
LDL-C (mmol/L)	2.90 ± 0.48	2.86 ± 0.67	3.07 ± 0.78	2.95 ± 0.72


*Values normally distributed are presented as the mean ± SD; values with skewed distribution are presented as medians with 25th–75th percentiles. NW, normal weight; OW/OB, overweight/obese; CRC, colorectal cancer; BMI, body mass index; SBP, systolic blood pressure; DBP, diastolic blood pressure; FBG, fasting blood glucose; TC, total cholesterol; TG, triglycerides; HDL-C, high-density lipoprotein cholesterol; LDL-C, low-density lipoprotein cholesterol. ^*a*^*P* < 0.05 compared with controls; ^*b*^*P* < 0.05 compared with NW+CRC patients.*

### Serum Levels of ATF3 and Irisin in CRC Patients and Controls

**FIGURE 1:**
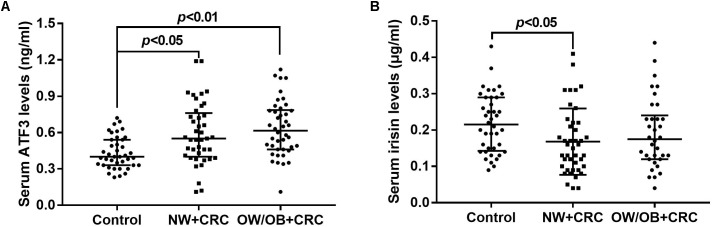
Serum ATF3 **(A)** and irisin **(B)** levels in CRC patients and healthy controls. All values are expressed as the median with 25th–75th percentile ranges. NW+CRC, colorectal cancer patients with normal weight; OW/OB+CRC, overweight/obese colorectal cancer patients; ATF3, activating transcription factor 3.

As shown in **Figure 1A**, ATF3 serum levels in the NW+CRC and OW/OB+CRC groups were 37.2 and 46.9% higher, respectively, than those in healthy controls (0.59 ± 0.04 vs. 0.43 ± 0.02 ng/mL, *P* < 0.05) (0.64 ± 0.23 vs. 0.43 ± 0.02 ng/mL, *P* < 0.01). No significant difference in serum ATF3 levels was observed between NW+CRC and OW/OB+CRC patients. Serum irisin levels in NW+CRC patients were 23.3% lower than those in healthy controls (0.17 ± 0.01 vs. 0.22 ± 0.01 μg/mL, *P* < 0.05) (**Figure 1B**). No significant difference in serum irisin levels was observed between NW+CRC and OW/OB+CRC patients (**Figure 1B**).

### *ATF3* and *FNDC5* mRNA Levels in sWAT and vWAT of NW+CRC and OB+CRC Patients

**FIGURE 2:**
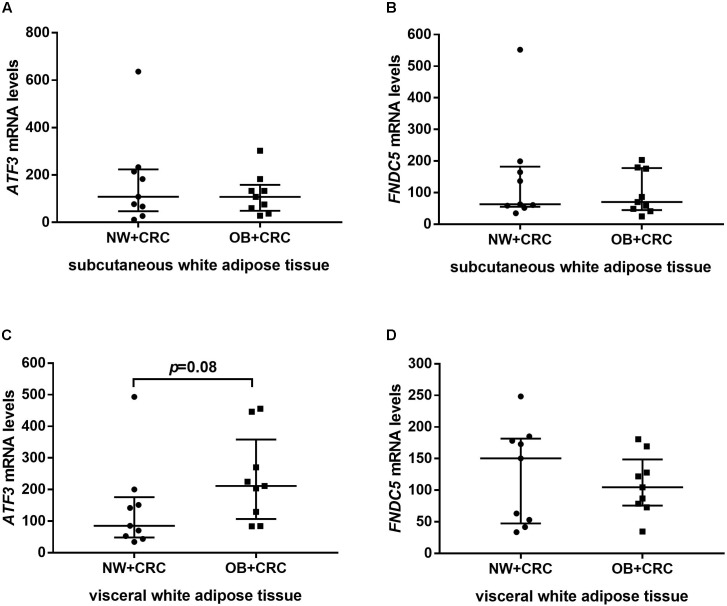
*ATF3* and *FNDC5* mRNA levels in sWAT **(A,B)** and vWAT **(C,D)** of NW+CRC and OB+CRC patients. All values are expressed as the median with 25th–75th percentiles. ATF3, activating transcription factor 3; FNDC5, fibronectin type III domain-containing protein 5; sWAT, subcutaneous white adipose tissue; vWAT, visceral white adipose tissue; NW+CRC, colorectal cancer patients with normal weight; OW/OB+CRC, overweight/obese colorectal cancer patients.

In addition, *ATF3* and *FNDC5* mRNA levels in sWAT and vWAT were also measured in nine NW+CRC and nine OB+CRC patients matched based on age and gender. General characteristics of the patients are summarized in **Supplementary Table S1**. As shown in **Figure 2C**, the *ATF3* levels in vWAT were increased by 65.6% in OB+CRC patients compared with those in NW+CRC patients; however, no statistically significant difference was detected (*P* = 0.08). No significant differences in the *ATF3* levels in sWAT or the *FNDC5* levels in sWAT and vWAT were noted between the two groups (**Figure 2**).

### Relationship Between Serum ATF3 and Irisin Levels and Clinical Parameters in CRC Patients and Controls

As listed in **Table 2**, serum ATF3 levels were positively correlated with BMI (*r* = 0.24, *P* < 0.05) and TG (*r* = 0.21, *P* < 0.05) but negatively associated with HDL-C (*r* = -0.37, *P* < 0.01) in all subjects. The positive correlation with BMI (*r* = 0.33, *P* < 0.05) and the negative correlation with HDL-C (*r* = -0.45, *P* < 0.01) were much larger in controls. Serum irisin levels were positively correlated with TG in all subjects (*r* = 0.24, *P* < 0.05) and in the CRC group (*r* = 0.32, *P* < 0.01).

**Table 2:** Bivariate correlation between serum irisin and ATF3 levels and other clinical parameters in CRC patients and controls.

	ATF3			Irisin		
		
	All (r)	Controls (r)	CRC (r)	All (r)	Controls (r)	CRC (r)
Age (year)	0.02	0.01	-0.15	-0.10	-0.24	0.05
Height (cm)	-0.06	-0.14	-0.02	-0.04	0.30	-0.22
Body weight (kg)	0.15	0.06	0.08	0.06	0.24	0.06
BMI (kg/m^2^)	0.24*^a^*	0.33*^a^*	0.09	0.08	0.16	0.16
SBP (mmHg)	0.01	-0.11	-0.07	-0.03	-0.04	0.05
DBP (mmHg)	-0.02	0.12	-0.04	-0.07	0.17	-0.02
FBG (mmol/L)	-0.03	-0.17	-0.12	0.08	0.10	0.16
TC (mmol/L)	-0.04	-0.13	0.02	0.13	-0.09	0.22
TG (mmol/L)	0.21*^a^*	0.26	0.15	0.24*^a^*	0.18	0.32*^b^*
HDL-C (mmol/L)	-0.37*^b^*	-0.45*^b^*	-0.13	0.11	-0.12	0.06
LDL-C (mmol/L)	0.09	0.19	0.07	0.08	-0.10	0.18


*Bivariate correlation was used to evaluate the relationship between irisin and ATF3 levels and other measured variables. ^*a*^*P* < 0.05; ^*b*^*P* < 0.01; CRC, colorectal cancer; ATF3, activating transcription factor 3; BMI, body mass index; SBP, systolic blood pressure; DBP, diastolic blood pressure; FBG, fasting blood glucose; TC, total cholesterol; TG, triglycerides; HDL-C, high-density lipoprotein cholesterol; LDL-C, low-density lipoprotein cholesterol.*

### Linear Regression Analysis for Variables Independently Related to ATF3 and Irisin Levels in Serum and WAT

A stepwise linear regression analysis model was used to further investigate the independent determinants for ATF3 and irisin levels in serum and WAT. As presented in **Table 3**, CRC (β = 0.29, *P* < 0.01) was an independent positive factor for serum ATF3 levels after adjusting for age, gender, BMI, TC, TG, SBP, DBP, FBG, and LDL-C. These findings were consistent with the data presented in **Figure 1A**, which revealed increased serum ATF3 levels in CRC patients. In addition, HDL-C (β = -0.25, *P* < 0.05) was an independent negative factor for serum ATF3 levels after adjusting for the same parameters. In contrast with the above association between ATF3 levels and CRC, the presence of CRC was independently negatively associated with serum irisin levels (β = -0.22, *P* < 0.05). These findings were consistent with the data presented in **Figure 1B**, which demonstrated reduced serum irisin levels in CRC patients. TG was independently associated with serum irisin levels after adjusting for the same parameters (β = 0.20, *P* < 0.05). Additionally, BMI was positively (β = 0.51, *P* < 0.05) related and SBP was negatively (β = -0.65, *P* < 0.01) related to *ATF3* mRNA levels in vWAT (**Table 3**).

**Table 3:** Variables independently related with ATF3 and irisin levels in serum and WAT based on multiple regression analysis (method stepwise).

	B (Beta coefficient)	Standard error	β (Standardized regression coefficient)	*P*
**Serum ATF3 (*R*^2^ = 0.20)**
CRC (0 or 1)	0.13	0.05	0.29	0.004
HDL-C	-0.19	0.07	-0.25	0.013
(Constant)	0.69	0.11		<0.001
**Serum irisin (*R*^2^ = 0.08)**
CRC (0 or 1)	-0.04	0.02	-0.22	0.019
TG	0.03	0.02	0.20	0.034
(Constant)	0.17	0.02		<0.001
***ATF3* mRNA in vWAT (*R*^2^ = 0.53)**
BMI	13.45	5.15	0.51	0.022
SBP	-8.55	2.58	-0.65	0.006
(Constant)	897.82	318.03		0.014


*Stepwise multiple regression analysis was used. Variables independently related with serum ATF3 and irisin levels were analyzed in all subjects, and variables independently related with *ATF3* mRNA levels in WAT were analyzed in nine NW+CRC and nine OB+CRC patients. Variables entered into multiple regression analysis but not included in the equation include age, gender, total cholesterol, diastolic blood pressure, fasting blood glucose, and low-density lipoprotein cholesterol.*

*vWAT, visceral white adipose tissue; 0; non-cancer; 1; colorectal cancer.*

### The Relationship of Serum ATF3 and Irisin Levels With CRC Risks

All participants were divided into three groups based on ATF3 tertiles (lowest: <0.41 ng/mL; median: 0.41–0.62 ng/mL; highest: ≥0.62 ng/mL). As shown in **Table 4**, the percentage of CRC increased as serum ATF3 concentrations increased across the tertiles (lowest: 15.5%, median: 21.6%, highest: 31.0%). After adjustment for age and gender, the probability of CRC in subjects with high ATF3 levels was greater by 13.2-fold compared with that in individuals with low levels (OR = 14.19, 95% CI 3.88–51.93, *P* < 0.01) (Model 1). The increased risk of CRC remained after further adjustment for BMI, FBG, SBP, and DBP based on Model 1 (Model 2, OR = 10.98, 95% CI 2.82–42.70, *P* < 0.01) and TC, HDL-C, TG, and LDL-C based on Model 2 (Model 3, OR = 23.32, 95% CI 3.96–137.53, *P* < 0.01). Additionally, serum irisin levels were also categorized into tertiles (lowest: <0.13 μg/mL; median: 0.13–0.23 μg/mL; highest: ≥0.23 μg/mL). In contrast with the association of serum ATF3 levels with CRC risks, the percentage of CRC decreased as serum irisin levels increased across the tertiles (lowest: 26.7%, median: 21.6%, highest: 17.2%). The CRC risk of participants with high irisin levels was lower by 77.0% compared with the risk for those with low serum irisin levels (OR = 0.23, 95% CI 0.08–0.66, *P* < 0.01) after adjustment for age and gender (Model 1). This decreased possibility of CRC remained after further adjustment for BMI, FBG, SBP, and DBP based on Model 1 (Model 2, OR = 0.17, 95% CI 0.05–0.56, *P* < 0.01) and TC, HDL-C, TG, and LDL-C based on Model 2 (Model 3, OR = 0.22, 95% CI 0.06–0.83, *P* = 0.026).

**Table 4:** Association of serum ATF3 and irisin levels with CRC risks based on unconditional logistic regression analysis.

Measurement	Tertiles (number of cases and controls)		
	
	Lowest OR (95% CI)	Median OR (95% CI)	Highest OR (95% CI)
**ATF3, range, ng/mL**	<0.41	≧0.41 to <0.62	≧0.62
Cases/controls	18/22	25/13	33/5
Percentage of CRC	15.5%	21.6%	31.0%
Univariate	1.00 (reference)	2.62 (1.04–6.62)	12.10 (3.55–41.31)
*P*-value		0.042	0.001
Model 1	1.00 (reference)	2.74 (1.04–7.24)	14.19 (3.88–51.93)
*P*-value		0.042	0.001
Model 2	1.00 (reference)	1.98 (0.65–6.01)	10.98 (2.82–42.70)
*P*-value		0.229	0.001
Model 3	1.00 (reference)	1.63 (0.42–6.28)	23.32 (3.96–137.53)
*P*-value		0.480	0.001
**Irisin, range, μg/mL**	<0.13	≧0.13 to <0.23	≧0.23
Cases/controls	31/7	25/14	20/19
Percentage of CRC	26.7%	21.6%	17.2%
Univariate	1.00 (reference)	0.40 (0.14–1.15)	0.24 (0.09–0.67)
*P*-value		0.090	0.006
Model 1	1.00 (reference)	0.43 (0.15–1.27)	0.23 (0.08–0.66)
*P*-value		0.127	0.006
Model 2	1.00 (reference)	0.34 (0.10–1.19)	0.17 (0.05–0.56)
*P*-value		0.090	0.004
Model 3	1.00 (reference)	0.25 (0.06–1.05)	0.22 (0.06–0.83)
*P*-value		0.058	0.026


*Multivariate odds ratios (OR) and 95% confident intervals (CI) from unconditional logistic regression models were used in the analysis.*

*Model 1: basic model, adjusted for age and gender.*

*Model 2: further adjusted for BMI, SBP, DBP, and FBG based on model 1.*

*Model 3: full model, further adjusted for TC (<5.18 and ≥5.18 mmol/L), TG (<1.7 and ≥1.7 mmol/L), HDL-C (<1.04 and ≥1.04 mmol/L), and LDL-C (<3.37 and ≥3.37 mmol/L) based on model 2.*

### Diagnostic Value of Serum ATF3 and Irisin Levels for CRC

**FIGURE 3:**
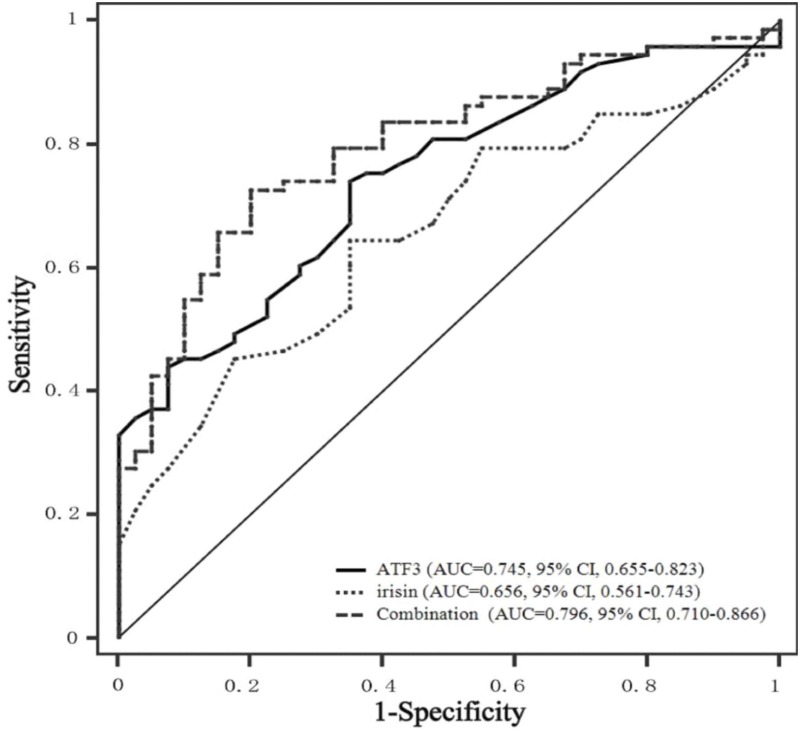
ROC curves of serum ATF3 and irisin levels. ROC curves were derived by plotting the relationship between the specificity and the sensitivity at various cut-off levels. ATF3, activating transcription factor 3; ROC, receiver operating characteristic; AUC, area under the curve.

The potential application of ATF3 and irisin for the discrimination CRC patients from healthy controls was evaluated by ROC curve analysis. As illustrated in **Figure 3**, ATF3 and irisin could discriminate CRC patients from healthy controls with ROC curve areas of 0.745 (95% CI, 0.655–0.823, *P* < 0.01) and 0.656 (95% CI, 0.561–0.743, *P* < 0.01), respectively. At the cut-off value of 0.46 ng/mL for ATF3, the sensitivity and specificity for the discrimination of CRC were 74.0 and 65.0%, respectively. At the cut-off value of 0.19 μg/mL for irisin, the sensitivity and specificity for the discrimination of CRC were 63.2 and 65.0%, respectively. When both ATF3 and irisin were included in the analysis, the ROC curve area was increased to 0.796 (95% CI, 0.710–0.866, *P* < 0.01) with 72.6% sensitivity and 80.0% specificity.

## Discussion

The *ATF3* gene is localized to the 1q amplicon, which is the most amplified chromosomal segment in human cancer (Rooney et al., 1999). Previous studies have reported significantly increased ATF3 expression in human CRC tissues (Wu et al., 2014; Yan et al., 2017). Studies performed by Wu et al. (2014) observed significantly increased ATF3 expression in human colon tissues compared with that in matched non-cancerous colon tissues. Similarly, Yan et al. (2017) also found increased ATF3 expression in human CRC tissues compared with that in adjacent normal tissues. To our knowledge, the present study is the first research to demonstrate that serum ATF3 levels in CRC patients with normal weight were significantly increased compared with those in healthy controls.

Our present study was the first to demonstrate that CRC was an independent factor associated with serum ATF3 levels after adjustment for age, gender, and other anthropometric and biochemical factors. Further unconditional logistic regression analysis demonstrated that the percentage of CRC was gradually increased as serum ATF3 levels increased from low to high tertiles. The probability of CRC in subjects with high ATF3 levels was considerably increased compared with the probability in those with low levels after adjustment for age and gender (Model 1). This increased risk of CRC remained after further adjusting for BMI, FBG, SBP, and DBP based on Model 1 (Model 2) and TC, HDL-C, TG, and LDL-C based on Model 2 (Model 3). These results suggested that ATF3 is a significant and independent risk factor for CRC. Our findings are supported by Wu et al.’s (2014) report, according to which the overexpression of ATF3 promoted the motility and invasiveness of human HT29 and CaCO2 colon cancer cell lines, whereas the knockdown of ATF3 expression using antisense ATF3 oligonucleotides inhibits adhesion and invasion in HT29 colon cancer cells (Ishiguro et al., 2000). Consistent with the results from colon cancer cell lines *in vitro*, studies performed by Ishiguro et al. (2000) *in vivo* revealed that mice inoculated subcutaneously with HT29 colon cancer cells and treated with the antisense oligonucleotide to knockdown ATF3 expression survived longer than did the control mice, owing to the inhibition of tumor growth and metastasis. These results together with our findings suggest that ATF3 may play an oncogenic role as it can promote the proliferation as well as the metastasis of CRC cell lines *in vitro* and *in vivo*. Thus, ATF3 serves as a significant risk factor for CRC. In contrast, studies performed by Hackl et al. (2010) demonstrated that ATF3 knockdown by RNAi in HCT116 colon cancer cells could increase subcutaneous tumor growth and promote hepatic metastasis as well as peritoneal carcinomatosis. The discrepancy might be attributed to the different genetic backgrounds of HT29 and HCT116 colon cancer cells.

In contrast to the elevated serum ATF3 levels in CRC patients, serum irisin levels were significantly lowered in CRC patients compared with those in healthy controls. Further analysis revealed that the percentage of CRC was gradually decreased as serum irisin levels increased from the low tertile to the high tertile. The CRC risk of participants with the high irisin levels was 23% of that with the low serum irisin levels after adjusting for age and gender (Model 1). This decreased probability of CRC remained after further adjusting for BMI, FBG, SBP, and DBP based on Model 1 (Model 2) and TC, HDL-C, TG, and LDL-C based on Model 2 (Model 3). Our results suggest that irisin might be a significant protective factor for CRC and is independent of other clinical anthropometric and biochemical items. Studies performed by Provatopoulou et al. (2015) demonstrated that serum irisin levels are significantly reduced in breast cancer patients compared with those in controls, and one unit increase in irisin levels leads to a reduction in the probability of breast cancer by approximately 90%. Further experiments conducted in cultured malignant breast epithelial cells *in vitro* found that irisin significantly decreases cell number and migration in MDA-MB-231 cells, and the viability of these cells is also reduced through the stimulation of caspase activity, leading to apoptotic death (Gannon et al., 2015). Similar results were obtained in cultured A549 and NCI-H446 lung cancer cells, demonstrating that irisin significantly inhibits the proliferation, migration, and invasion of lung cancer cells via suppression of the epithelial-to-mesenchymal transition (Shao et al., 2017). All these findings together with our present results suggest that irisin might be a new diagnostic indicator of the presence of cancer. However, in disagreement with these results, studies performed by Aydin et al. (2016) revealed a significant increased irisin immunoreactivity in colon adenocarcinoma tissue compared with that in normal colon tissues in Turkish people. *In vitro* studies performed by Moon and Mantzoros (2014) showed that irisin at both physiological and pharmacological concentrations has no effects on cell proliferation, cell adhesion, and/or colony formation in HT29 and MCA38 colon cancer cell lines. Considering that these are the only two currently available studies assessing the role of irisin in colon cancer, further studies are highly needed to elucidate the effects of irisin in the occurrence and development of CRC.

Finally, the optimal cut-off points of serum ATF3 and irisin for the diagnosis of CRC based on ROC analysis were also evaluated for clinical practice. Our results indicated that serum ATF3 and irisin could effectively discriminate CRC patients from healthy individuals within ROC curve areas of 0.745 and 0.646, respectively. The optimal cut-off values of ATF3 and irisin are 0.46 ng/mL and 0.19 μg/mL, respectively. In addition, the combination of ATF3 and irisin exhibits improved diagnosis value accuracy with ROC curve areas of 0.796, which is greater than that noted for either ATF3 or irisin alone. These results indicated that both ATF3 and irisin may be used as potential serum diagnostic markers for CRC, and the combination of ATF3 and irisin could provide improved diagnostic results.

In addition, we found that ATF3 expression in vWAT tends to be increased in obese CRC patients compared with the expression in those with normal weight, and the BMI was positively related with the ATF3 levels in serum and vWAT. Consistent with our results in CRC patients, studies performed by Kim et al. (2006) demonstrated that the *ATF3* mRNA levels in WAT are also drastically upregulated in both *ob/ob* and *db/db* mice. Two previous studies have reported that ATF3 inhibits 3T3-L1 preadipocyte differentiation (Jang et al., 2012; Jang and Jung, 2014). Of note is that the number of preadipocytes capable of undergoing differentiation is reduced in obese patients and is negatively correlated with the BMI (Wood et al., 2009). Therefore, ATF3 may play a role in impaired adipocyte differentiation in obesity such that excess lipids cannot be stored adequately in the WAT, thus inducing obesity and its related metabolic disorders.

Our data also demonstrated that a positive correlation exists between serum irisin and TG levels, and that TG is independently associated with serum irisin levels after adjusting for the general clinical and laboratory parameters. Consistent with our results, Shoukry et al. (2016) also observed the positive correlation between circulating irisin and TG in type 2 diabetes patients. However, a negative correlation between irisin and TG has been reported in overweight/obese children and adolescents aged 6 to 10 years old (Suk et al., 2018). Alternatively, no correlation between irisin and TG was reported in pregnant women with gestational diabetes mellitus (Ural et al., 2016). Thus, given that our present study is the first to demonstrate an association between irisin and TG in CRC patients, the relationship should be further assessed in future studies.

Taken together, our present study first found that serum ATF3 levels were significantly increased and irisin levels were reduced in Chinese CRC patients. CRC was independently associated with circulating ATF3 and irisin levels. Subjects with increased ATF3 and reduced irisin levels were more likely to have CRC after adjusting for age, gender, BMI, and other biochemical variables. ATF3 and irisin may be used as potential serum diagnostic markers for CRC. However, our present study was conducted in small samples of Chinese people. Further studies must be performed in larger samples and other ethnic populations.

## Author Contributions

HZ designed the experiments and revised the primary manuscript. ML analyzed the data and wrote the primary manuscript. NZ performed molecular biological experiments. HP, GL, NL, LW, HY, and KY collected the clinical materials and serum samples and completed the clinical and biochemical parameter measurements. FG designed the experiment, supervised the entire study, and revised the primary manuscript.

## Conflict of Interest Statement

The authors declare that the research was conducted in the absence of any commercial or financial relationships that could be construed as a potential conflict of interest. The reviewer SK and handling editor declared their shared affiliation at the time of the review.
