# Supplementary material for: Serum and Adipose Tissue mRNA Levels of ATF3 and FNDC5/Irisin in Colorectal Cancer Patients With or Without Obesity
Source: Front Physiol. 2018 Sep 10;9:1125. doi: 10.3389/fphys.2018.01125 (PMC6140752; doi:10.3389/fphys.2018.01125)
Supplement: Supplementary file 1 [file Table_1.DOCX]

**Table S1 The general characteristics of the 9 NW+CRC and 9 OB+CRC patients**

| **Characteristics** | **NW+CRC**  **(n=9)** | **OB+CRC**  **(n=9)** |
| --- | --- | --- |
| Gender (M: F) | 5/4 | 5/4 |
| Age (y) | 64.78±5.93 | 64.67±10.05 |
| Height (cm) | 166.56±6.37 | 165.00±7.35 |
| Body weight (kg) | 59.89±5.11 | 87.67±11.69 ^a^ |
| BMI (kg/m^2^) | 21.56±0.81 | 32.04±1.63 ^a^ |
| SBP (mmHg) | 121.50±11.89 | 126.89±10.19 |
| DBP (mmHg) | 69.88±9.75 | 77.56±12.22 |
| FBG (mmol/L) | 5.09±0.42 | 6.01±2.24 |
| TC (mmol/L) | 4.65±0.74 | 5.06±0.99 |
| TG (mmol/L) | 1.29±0.66 | 1.31±0.41 |
| HDL-C (mmol/L) | 1.07±0.25 | 1.07±0.24 |
| LDL-C(mmol/L) | 2.99±0.66 | 3.16±0.58 |

Values are mean ± SD; NW+CRC, colorectal cancer patients with normal weight; OB+CRC, colorectal cancer patients with overweight/obese; BMI, body mass index; SBP, systolic blood pressure; DBP, diastolic blood pressure; FBG, fasting blood glucose; TC, total cholesterol; TG, triglycerides; HDL-C, high-density lipoprotein cholesterol; LDL-C, low-density lipoprotein cholesterol. ^a^*p*<0.01 compared with NW+CRC group.
